# Three-Dimensional Plotted Calcium Phosphate Scaffolds for Bone Defect Augmentation—A New Method for Regeneration

**DOI:** 10.3390/jpm13030464

**Published:** 2023-03-02

**Authors:** Matthias C. Schulz, Stefan Holtzhausen, Berthold Nies, Sascha Heinemann, David Muallah, Lysann Kroschwald, Kristin Paetzold-Byhain, Günter Lauer, Philipp Sembdner

**Affiliations:** 1 Department of Oral and Maxillofacial Surgery, University Hospital Tübingen, Eberhard Karls Universität Tübingen, Osianderstraße 2-8, 72076 Tübingen, Germany; 2 Department of Oral and Maxillofacial Surgery, University Hospital “Carl Gustav Carus”, Technische Universität Dresden, Fetscherstraße 74, 01307 Dresden, Germany; 3 Institute of Machine Elements and Machine Design, Chair of Virtual Product Development, Technische Universität Dresden, 01062 Dresden, Germany; 4 INNOTERE GmbH, Meissner Str. 191, 01445 Radebeul, Germany

**Keywords:** calcium-phosphate scaffold, computer aided design, computer aided manufacturing, sinus floor augmentation

## Abstract

For sinus grafting, different methods and materials are available. One possible shortcoming of particulate bone grafts is either overfilling or augmenting the planned implant area insufficiently. To overcome this risk and to determine the implant position prior augmentation, we present an approach using three-dimensional printed scaffolds. A patient with a remaining anterior dentition and bilateral severely atrophied posterior maxilla was seeking oral rehabilitation. The cone beam computed tomography (CBCT) showed residual bone heights between one and two millimeters. Following the three-dimensional reconstruction of the CBCT data, the positions of the implants were determined in areas 16 and 26. Three-dimensional scaffolds adapted to the topography of the sinus were virtually designed and printed using a calcium phosphate cement paste. Bilateral sinus floor augmentation applying the printed scaffolds with an interconnecting porosity followed. After nine months, a satisfying integration of the scaffolds was obvious. At the re-entry, vital bone with sufficient blood supply was found. One implant could be placed in positions 16 and 26, respectively. After five months, the implants could be uncovered and were provided with a temporary denture. The application of three-dimensionally printed scaffolds from calcium phosphate cement paste seems to be a promising technique to graft the severely atrophied posterior maxilla for the placement of dental implants.

## 1. Introduction

The grafting of large or complex shaped bony defects is still a challenge for oral and maxillofacial surgeons. However, grafting is frequently required in order to enable oral rehabilitation using dental implants. Recently, the application of three-dimensional printed scaffolds has become an emerging technique for bone augmentation in the oral and maxillofacial area [1,2]. Based on a three-dimensional data set, e.g., radiographical image data from a cone beam computed tomography (CBCT), scaffolds are virtually designed and generated from different materials. Thus, it is possible to reconstruct even defects of a complex geometrical shape [3]. The most frequently used origins for scaffolds in oral bone regeneration are allogenic, xenogenic or alloplastic [4]. While allogenic and xenogenic materials have pre-set properties, e.g., porosity and absorbability, these properties are adjustable in alloplastic materials. A varying porosity or absorbability potentially influences cell migration and proliferation and, thus, a more rapid ingrowth of bone into the scaffold [3]. Considering the absorbability, it was observed that a fast resorbing calcium phosphate bone substitute was directing the structural alterations of the host bone to vital bone tissue in a radiographical study [5]. It was stated that the optimal degradation rate of the scaffold is similar to the rate of osteogenesis [6]. Thus, the scaffold’s structure would be replaced by new bone. Furthermore, porosity and pore size are crucial parameters influencing the attachment, differentiation and proliferation of cells [3,7]. Ideally, an increased porosity of the material enables cell proliferation via interconnective pores. On the other hand, a higher pore size or porosity decreases the mechanical resistance [3,7]. Furthermore, the strand orientation and tortuosity of the pore channels might influence the mechanical stability and permeability [8,9]. A higher permeability supports cell migration and, thus, promotes bone tissue regeneration better than straight microchannels [9]. Currently, there is a wide variety of materials used for three-dimensional printing of scaffolds for bone tissue engineering ranging from polymers, metals, ceramics and hydrogels to carbon-based nanomaterials [10,11]. A disadvantage might be the necessity to process these materials at higher temperatures, e.g., metals and ceramics. Other materials suffer from a rapid loss of tensile strength during the degrading process, e.g., polylactides [12]. In order to overcome these shortcomings of single materials, composites of various materials are used for scaffold processing combining the desired properties [13].

A material having shown its suitability for the three-dimensional printing of scaffolds for bone regeneration is calcium phosphate cement (CPC) [14]. Calcium phosphate cements are defined as hydraulic cements consisting of one or more calcium orthophosphate powders and a liquid phase [15]. CPC can be of a pasty consistency and cures at considerably low temperatures such as room or body temperature at a nearly neutral pH-value [16]. The material is bioactive and osteoconductive which means that it provides a scaffold for the migration and proliferation of osteoblasts [17]. Additionally, it shows a slow biodegradability resulting in a high volume stability over years [18]. An advantage of CPC is its moldability and printability enabling the modification of the basic material [17,19]. Furthermore, calcium phosphate cements can be used as a carrier for certain agents in order to stimulate bone regeneration [20,21]. Due to these properties, the material has shown its suitability for three-dimensional printing for clinical applications in several studies yet [3,5,22,23].

Sinus floor augmentation is an established and predictable procedure to graft bone in the posterior maxilla [24]. A variety of grafting methods and materials is available [25]. The ideal grafting material offers volume stability, offers sufficient filling of the subantral space and enables new bone ingrowth [26,27]. Furthermore, it should allow the insertion of dental implants with a sufficient primary stability after the healing period [28]. Rarely, the dislocation of substitute particles might occur which increases the postoperative morbidity [29]. One possibility to overcome this potential dislocation might be the application of scaffolds for sinus floor augmentation. In a case series, three-dimensionally milled scaffolds made from hydroxyapatite were used for bilateral sinus floor augmentation [30]. However, when inserting hydroxyapatite scaffolds from coralline origin, no adjustment of the porosity or mechanical stability is possible. Thus, scaffolds made from calcium phosphate cement seem to be a promising option for the augmentation of the maxillary sinus. 

To the best of our knowledge, this is the first case where three-dimensional printed scaffolds made from calcium phosphate cement paste with interconnecting porosity were used for sinus grafting. The aim of the presented case was to examine the proof of principle for the clinical application of three-dimensional printed calcium phosphate cement scaffolds for sinus grafting.

## 2. Case Presentation

The case of a 62-year-old male patient seeking a better retention of his partial removable denture in the maxilla is presented. His medical history contained an atrial fibrillation and a cardiac arrhythmia. The routine medication was composed of rivaroxaban 20 mg daily. There were no allergies.

The initial intraoral findings showed a remaining dentition in the maxilla containing the anterior teeth from canine to canine and the second premolar on the right. A horizontal bone loss up to the median third of the tooth roots was clinically obvious. Furthermore, gingival recessions on the facial site of the anterior teeth were detected. The left lateral incisor and canine were provided with dental crowns including a retainer for the removable partial denture. The removable partial denture was replacing the first premolar and the first and second molar on the right side. On the left side, both premolars and molars were replaced. The base of the denture was not covering the palatal area. Retentions were fixed on the second right premolar and on the left canine. In the mandible, only the first molars on both sides were missing without being replaced. The probing depths were slightly increased. The mucosal tissues showed no abnormalities.

**Figure 1:**
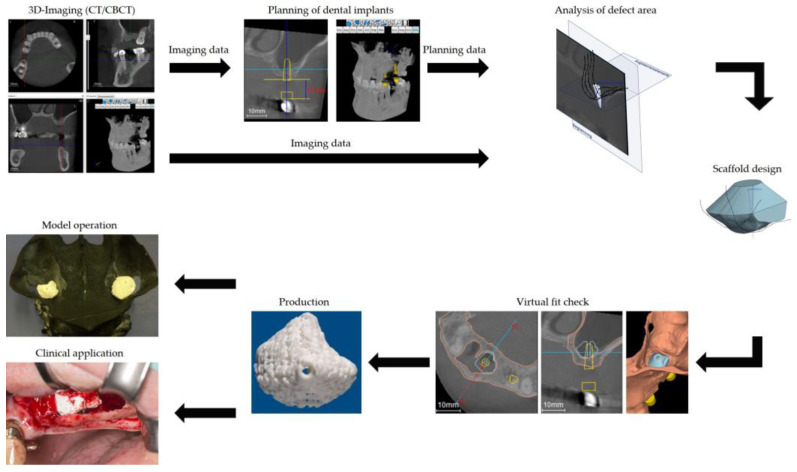
Schematic overview of the workflow in clockwise direction starting top left. Acquisition of the three-dimensional radiographic data (upper left). Planning of the position of the dental implants with yellow contour indicating the planned implant and the corresponding sleeve (upper center). Analysis of the defect size and topography (upper right). Virtual design of the scaffold (center right). Virtual check of the designed scaffold with yellow contour indicating the planned implant and the corresponding sleeve, light blue contour indicating the planned scaffold and brown contour indicating the pristine bone in the transversal plane; coronary plane is indicated by the red arrows (lower right). Manufacturing of the scaffolds (lower center). Check of the designed scaffold in a three-dimensional model of the defect situation (center left). Clinical application of the scaffold in the patient (lower left).

Following the clinical examination, a cone beam computed tomography (Accuitomo, J. Morita Corporation, Osaka, Japan) of the maxilla was performed. The parameters were as following: field of view: 80 × 80 mm, voxel size: 0.16 mm, tube voltage: 87 kV, current: 8 mA. The data were saved as Digital Imaging and Communications in Medicine (DICOM) format. The radiographical findings elucidated a vertical bone dimension of 2–3 mm bilaterally in the posterior maxilla. On both sides, the maxillary sinuses showed no signs of inflammation or residual roots. The patient was informed that the insertion of dental implants would require a bilateral sinus augmentation procedure. Furthermore, the potential methods for sinus floor augmentation were discussed extensively. Figure 1 shows an overview of the workflow.

**Figure 2:**
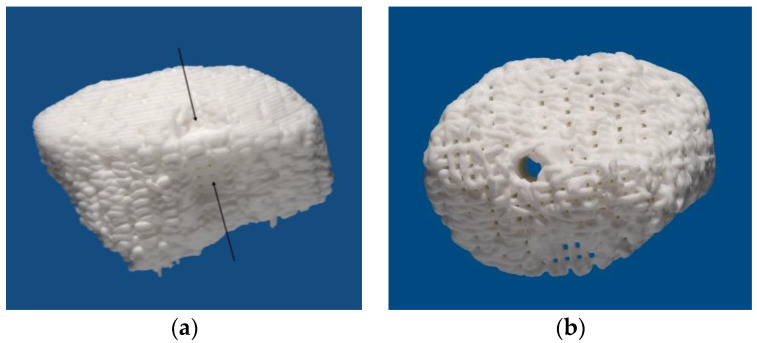
Scaffold for grafting the left maxillary sinus. (**a**) Lateral aspect with black arrows depicting the grooves for the tweezers. (**b**) The structure of the calcium phosphate strains and the cavity for the fixation screws are clearly visible.

After the informed consent of the patient, the design of the scaffolds was started. In the sense of backward planning, the planning of the prosthetic structure was first performed using the Organical Dental Implant software (Organical CAD/CAM GmbH, Berlin, Germany). One dental implant with a length of approximately 10 mm and a diameter of 4.5 mm was planned for the replacement of the first molar on each side. Based on this planning, the design of the scaffold followed. For this purpose, the position and dimensions of the scaffolds that were planned to be inserted were determined in consultation with the surgeons. Digital models and screenshots were used to visualize the desired regions. The models and screenshots were created applying the Organical Dental Implant software (Organical CAD/CAM GmbH, Berlin, Germany). Subsequently, the design of three different alternatives considering the shape of the scaffold was carried out. The design was created using SolidWorks software of Dassault Systèmes (SolidWorks Deutschland GmbH, München, Germany) in combination with CTinA software, a framework developed by the Chair of Virtual Product Engineering (VPE) with a software solution for processing CT data for virtual product development. CTinA was used to process the data during the design process, such as local segmentation of regions of interest, and to derive design feature elements from the imaging data. This data and information were then transferred to the SolidWorks design program via a software interface. The designed alternatives differed in the spatial shape, the height and the geometric design of the cranial side since this surface has no bone contact but is in contact to the Schneiderian membrane. Thus, a design had to be found that would not damage this vulnerable structure. A close cooperation between engineers and surgeons was required. Following an interdisciplinary discussion, the final design of the scaffolds was found. The scaffolds had a height of approximately 10 mm. Thus, the planned dental implant would be fully inserted into the scaffold structure. In addition, functional elements were integrated, e.g., a cavity for inserting the fixation screw having a diameter of 2 mm. The cavity was positioned along the planned axis of the dental implants. The transfer of the planning data from the Organical Dental Implant software to the CTinA software was performed via a software interface developed by VPE. Furthermore, pockets with a size of approximately 2 × 3 mm were integrated laterally enabling simple handling with tweezers during surgery (Figure 2).

Subsequently, the data were sent to the manufacturer (INNOTERE GmbH, 01445 Radebeul, Germany) for the production of the scaffolds.

The manufacturing process of the scaffolds was completely based on the workflow similar to the CE-labeled class 3 medical device product family. Therefore, the validity of the applied materials, equipment and processes was proven. The calcium phosphate cement paste was prepared as described previously [31]. The three-dimensional printing was carried out using a custom-designed 3D printer in a controlled clean room environment. The common xyz-table was modified with a VIEWEG pneumatic system (VIEWEG GmbH - Dosier- und Mischtechnik, Kranzberg, Germany) working with sterile air to extrude the cement which was filled into 10cc Nordson cartridges (Nordson, Westlake, OH, USA). The documentation was embedded in a DIN EN ISO 13485 Quality Management System. Before use, the substrate structures were chemically sterilized. The reference between the dosage needle (steel, 330 µm diameter, Nordson, Westlake, OH, USA) and the support structure was established. The three-dimensional printing was carried out at a head speed of up to 8 mm/s. The scaffold design was based on a typical strand distance of double strand diameter resulting in a gap similar to the strand diameter. The meandering layers with an orthogonal direction and an ABAB layer pattern resulted in a pore size of 330 µm size, which were interconnecting in all directions. The samples were exposed to a high relative humidity to induce cement setting over seven days. The hardened scaffolds were removed from the support structures and rinsed in acetone for carrier liquid removal and drying. The dried scaffolds were double-packed in Tyvek bags and Co-60 gamma-irradiated at 35 kGy (STERIS Synergy Health, Radeberg, Germany). The quality inspection before final release included analysis for overall shape, weight, porosity and compressive strength.

Next, a model of the patient’s maxilla was printed in order to evaluate the suitability of the scaffolds and to determine the extent of the access cavities. Before performing the clinical surgery, a model surgery was carried out using the resin model. The resin model was based on a segmentation performed in the Organical Dental Implant software. The extracted surface model was then prepared in the software Geomagic Studio 2012 (3D Systems GmbH, Moerfelden-Walldorf, Germany). The model was three-dimensional printed on an FDM Vantage S printer (Stratasys GmbH, Rheinmünster, Germany). The material used was PC-ISO, a biocompatible polycarbonate material (Stratasys GmbH, Rheinmünster, Germany).

**Figure 3:**
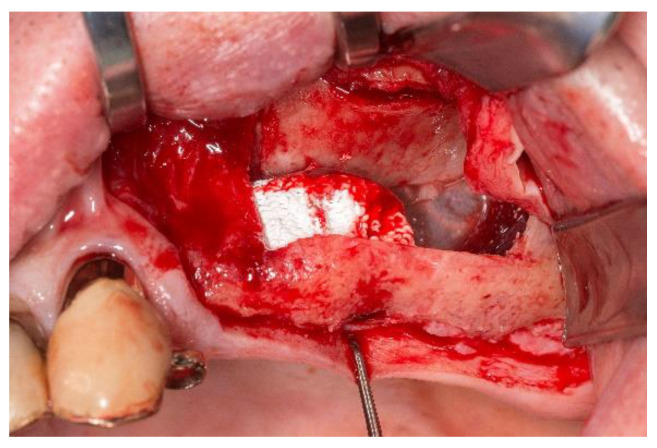
The intra-operative situs after fixation of the scaffold in the left maxillary sinus with a fixation screw on the crestal bone. The access bone lid is deflected into the maxillary sinus.

Next, the bilateral sinus floor elevation was performed using the calcium-phosphate scaffolds. All surgeries were performed according to the department’s standard in local anesthesia using Ultracain DS (Sanofi Aventis Deutschland, Frankfurt, Germany). Following a crestal incision with a mesial relief incision, a mucoperiosteal flap was elevated. The access cavities were prepared using a piezosurgery device (Piezosurgery^®^ 3, mectron Deutschland Vertrieb, Köln, Germany). The resulting bone lid was deflected cranially and remained pedicled on the Schneiderian membrane (Figure 3). On the left side, a small rupture of the Schneiderian membrane occurred which was sutured with absorbable material (Vicryl 5-0, Johnson & Johnson Medical GmbH, Ethicon Deutschland, Norderstedt, Germany). Subsequently, the calcium-phosphate scaffolds were placed in the sub-antral space and fixed with each one micro screw (1.5 × 7 mm; Gebrüder Martin, Tuttlingen, Germany). The scaffold in the right maxilla was measuring approximately 10 × 10 × 8 mm and in the left maxilla 10 × 14 × 8.5 mm, respectively. After the repositioning of the mucoperiosteal flap, the wound closure was performed using non-absorbable sutures (Prolene monofil 5-0, Johnson & Johnson Medical GmbH, Ethicon Deutschland, Norderstedt, Germany). Ibuprofen was administered as analgesic. The postoperative behavioral instructions contained the application of cool packs, a liquid diet and the use of decongestant nasal spray. In order to examine the correct position of the scaffolds, a postoperative CBCT was performed.

After a healing period of nine months, the re-entry for implant insertion was performed in local anesthesia. After the bilateral crestal incision and the elevation of a mucoperiosteal flap, the micro screws could be removed without problems. In order to obtain a tissue biopsy for histological analysis of the augmented site, the implant cavities in regions 16 and 26 were prepared using a trephine drill of 3 mm outer diameter (Hager & Meisinger, Neuss, Germany) for the pilot cavity. Subsequently, the implant cavities were prepared according to the manufacturer’s protocol, and each one implant of 11.5 mm length (SIC invent AG, Basel, Switzerland) was inserted in regions 16, 24 and 26. All implants achieved a sufficient primary stability. The mucoperiosteal flaps were repositioned, and the wound closure was performed with non-absorbable sutures (Prolene monofil 5-0, Johnson & Johnson Medical GmbH, Ethicon Deutschland, Norderstedt, Germany).

The histological samples were fixed in 4% formaldehyde for 48 h. After dehydration in a graded series of ethanol, the specimens were embedded in methylmethacrylate (Technovit 9100 NEU, Heraeus Kulzer, Wehrheim, Germany). Longitudinal sections of 100 µm thickness in oro-vestibular direction were produced using Donath’s sawing and grinding technique [32]. The central section of each specimen was chosen for evaluation. After polishing, the sections were stained using the Masson-Goldner trichrome staining. Subsequently, the specimens were imaged using a light microscope (Olympus BX 61, Olympus Deutschland, Hamburg, Germany). Using an automatic scanning table (Märzhäuser, Wetzlar, Germany), six images per trephine core were scanned with a 4 × 10-fold magnification and fused to one image. The images were analyzed histologically regarding bone formation and foreign body reactions.

After a healing period of five months, the second stage implant surgery for uncovering the implants followed. In local anesthesia (Ultracain DS, Sanofi Aventis Deutschland, Frankfurt, Germany), a palatal incision was performed, and the mucosal flaps were deflected to uncover the implants. Healing abutments (SIC 3.3 × 4 mm and 3.3 × 3 mm, SIC invent AG, Basel, Switzerland) were fixed. Wound closure again was performed with non-absorbable sutures (Prolene monofil 5-0, Johnson & Johnson Medical GmbH, Ethicon Deutschland, Norderstedt, Germany). Postoperatively, a panoramic radiograph was carried out to examine the osseointegration of the implants.

Subsequently, the abutments of the implants were integrated in the partial removable denture to increase the number of support structures. Thus, a sufficient retention of the prosthesis was achieved.

## 3. Outcome

### 3.1. Handling of the Scaffolds and Clinical Outcome

During the surgery, the manageability of the scaffolds was sound. Due to the grooves on the vestibular side, the scaffolds could be handled and hold in place during their fixation by using a pair of tweezers (Figure 1). From the consistency, the scaffolds appeared brittle. Thus, the screws used for fixation of the scaffolds had to be of a reduced diameter in order to avoid cracking of the scaffolds.

After the insertion of the scaffolds, a slight swelling was obvious bilaterally. However, the swelling appeared to be soft indicating a post-operative edema. The wound healing was without abnormalities; no dehiscences or fistula were detectible. The patient reported no intense pain. No clinical signs of a sinusitis or nasal congestions were obvious. Thus, the sutures could be removed after 14 days. The healing period of the scaffolds was without any abnormal finding.

**Figure 4:**
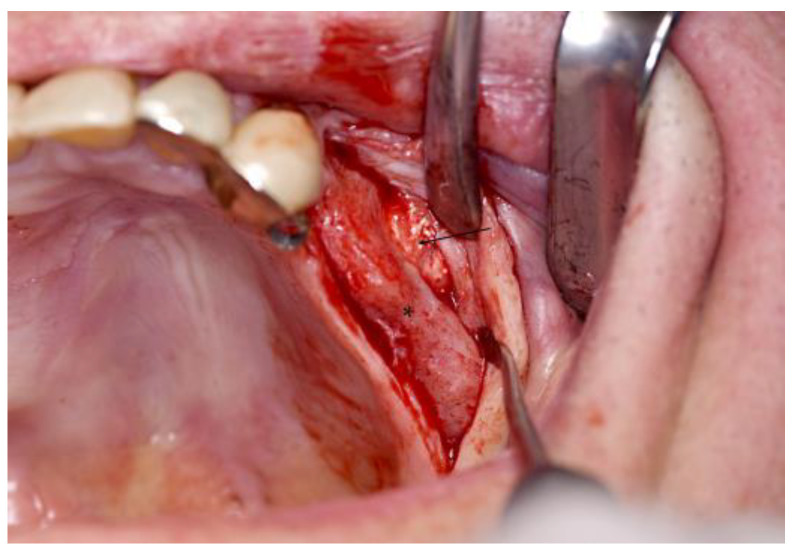
Clinical outcome. At the time of re-entry for implant insertion, the scaffolds showed a sufficient osseointegration without any signs of inflammation. The fixation screw was covered by bone (*). The structure of the scaffold is visible at the lateral aspect of the sinus wall (black arrow).

After a healing period of nine months, the bony sites showed no sign of osteolysis. The fixations screws were partly covered by bone. The structure of the scaffolds with obvious ingrowth of vital bone appeared less brittle compared to the insertion of the scaffolds (Figure 4). When obtaining the trephine cores, the material of the scaffolds appeared more solid than the pristine bone. The implant insertion was possible with a sufficient primary stability. No rupture of the Schneiderian membrane could be detected. After 14 days, the wounds were without any sign of swelling or inflammation. Thus, the sutures could be removed. During a check-up appointment three months after implant insertion, the cover screw of the implant in the right maxilla was exposed. However, the surrounding mucosa showed no signs of inflammation.

At the re-entry for the uncovering of the implants, the implants were clinically sufficiently integrated and showed no sign of inflammation or loosening. After insertion of the healing abutments, no abnormalities could be observed. Therefore, the implant abutments could be sufficiently used as additional support for the partial dental prosthesis in the maxilla.

### 3.2. Radiographical Outcome

**Figure 5:**
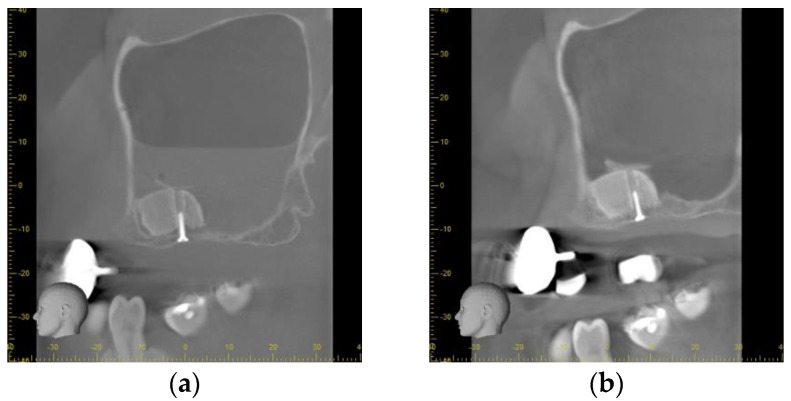

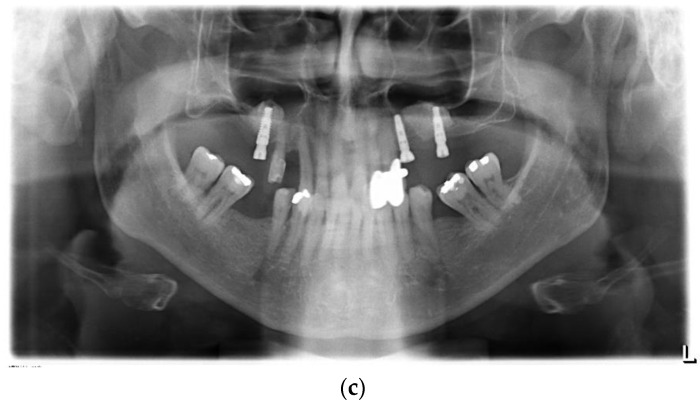
Radiographical outcome (**a**) Sagittal plane of the left maxillary sinus directly after augmentation. An air-fluid-level of blood is visible after a small perforation of the Schneiderian membrane. The scaffold is tightly fixed to the host bone by the screw. (**b**) Sagittal plane of the left maxillary sinus prior to implant placement. The scaffold is showing sufficient osseointegration. The bone lid is visible cranial of the scaffold. No sign of inflammation or mucosal swelling is detectable in the sinus. (**c**) Panoramic radiograph after implant uncovering. A sufficient osseointegration of the scaffolds and the dental implants without any signs of peri-implant osteolysis is obvious. No sign of inflammation of the maxillary sinuses is detectable.

Directly after the insertion of the bone substitute, the cone beam computed tomography confirmed the correct position of the scaffolds according to the planned position. On the left side, a slight gap between the palatal bone wall and the scaffold was observed. The bone lids used to access the sinuses were located on the cranial site of the scaffolds. As a consequence of the small perforation of the Schneiderian membrane, a level of liquid was obvious in the left maxillary sinus whereas on the right side a slight swelling was observed (Figure 5a).

After the healing period of 9 months, no signs of inflammation were found in both maxillary sinuses. The scaffolds showed a sufficient integration into the host bone without any sign for osteolysis or sequestration. The bone lids were still located on the cranial site of the scaffolds appearing similar to Underwood septa. No sign of loosening or dislocation of the fixations screws was found (Figure 5b).

Following the uncovering of the implants, the panoramic radiograph showed a sufficient osseointegration of all implants. No sign of an inflammation of the maxillary sinuses was found. The scaffolds were found to be sufficiently osseointegrated. No osteolytic areas could be observed (Figure 5c).

### 3.3. Histological Outcome

**Figure 6:**
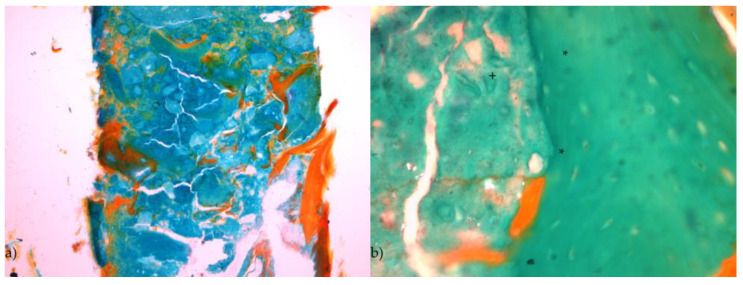
Histologic specimen of region 26 (Masson-Goldner staining). (**a**) Trephine core showing the close contact between the vital bone and the scaffold with a denser structure in the cranial area of the core (Magnification: 10-fold). (**b**) Detail showing the close contact between bone (*) and scaffold material (+) without a gap (Magnification: 40-fold).

The trephine core showed dense structures of the bone substitute in close contact to mature bone. In the contact area between the bone substitute and the mature bone, zones of osteoid deposition could be found. At the margins of the trephine core, formations of connective tissue were obvious which were proceeding with thin extensions to the center. No formation of inflamed or necrotic tissue could be found in the trephine core (Figure 6a).

When looking at the contact area between the bone substitute and the bone with an increased magnification, osteons could be found. No gap between the substitute and the new formed bone was obvious indicating a sufficient integration of the substitute material (Figure 6b). Signs of resorption of the substitute material could not be observed at the time of implant placement. 

## 4. Discussion

In the presented case, three-dimensionally printed calcium phosphate scaffolds were used to graft the maxillary sinus bilaterally in order to obtain a sufficient bone volume for the insertion of dental implants. Clinically, bone grafting and implant insertion were not showing any sign of complications. Besides post-operative swelling and pain which were easily treatable with ibuprofen, no side-effects could be observed.

An advantage of the application of scaffolds is the precise fitting into the defect. Thus, a dislocation of the grafting material, which might occur when no membrane is used, seems unlikely [29]. However, considering the printing tolerance, the error in our case is much higher compared to another similar set-up where a three-dimensional milled scaffold was used. In the examination of Mangano et al., a cutting accuracy of 25 µm was stated [30]. In the present case, the three-dimensional printing of the scaffolds was carried out with a printing accuracy of 330 µm limited by the diameter of the printing needle. However, when adding the errors of the workflow, a higher inaccuracy will occur. The voxel size of the CBCT is stated with 0.16 mm cumulating to an effective error of 0.32 mm. Additionally, an offset of 0.1 mm for the printing was setup in order to avoid a poor fit due to, e.g., bone spikes. Thus, in the worst case a total workflow error of 0.75 mm is possible. However, the clinical impact of this error is questionable as the scaffold is inserted by a surgeon and a positioning error of less than 1 mm can be considered as sufficient [33]. Moreover, the scaffold was fixed tightly to the adjacent bone by a fixation screw enabling a close bone-scaffold-contact and, thus, a sufficient healing. Furthermore, the fixation with the screw keeps the scaffold in place. One shortcoming in handling the scaffold was the brittle consistency, potentially increasing the risk of cracking when tightening the fixation screws. However, by printing the cavity for the screw in a slightly oversized dimension, this flaw was overcome in the presented case. No crack or fracture of the scaffolds was detected during the surgery. One possible weak point might be the accessibility of the fixation screw, certainly when the grafting area is in the posterior maxilla, or the mouth opening is hampered. In this case, a sufficient fixation of the scaffold might not be possible. However, this issue can be solved by angulating the screw cavity which is simply feasible as the three-dimensional, virtual planning offers a great freedom in the design of geometry and shape. Another advantage might be the possibility to adjust the pore size of the scaffold material. The pore has a crucial impact on cell migration into the scaffold and proliferation [3,7]. By varying the pore size, an increased cell migration and proliferation and, thus, a faster bone ingrowth into the scaffold might be possible. This could allow reduced the healing time and, therefore, a shortened treatment period for the patient. When applicating customized scaffolds from xenogeneic origin, the pore size is pre-set and cannot not be adjusted [30]. This might hamper cell migration and osseintegration of the scaffolds. Furthermore, an adaptation to varying loads in different regions of the oral and maxillofacial area is not possible. This might be a disadvantage as scaffolds might not be suitable for grafting in the mandible and maxilla. Herein, there might be an advantage when using three-dimensional printing, where the pore size can be adjusted to biological and mechanical requirements which are differing in non-load-bearing regions, e.g., maxillary sinus and load-bearing areas in the mandible [3]. Considering the varying stress, additional tests for scaffolds seem to be important in order to analyze the long-term stability certainly of absorbable scaffolds [34]. In an in vitro setting, it was found that porous magnesium scaffolds show an increased fatigue when being immersed in simulated body fluids [34]. However, the applied scaffolds were showing a much higher pore size with 800 µm compared to the 330 µm in our case. Furthermore, the sub-antral space can be considered as non-load bearing.

A recent histomorphometric analysis of human biopsies demonstrated that the packing density might affect the bone regeneration in sinus floor augmentation using particulate material [35]. It was shown that high packing density led to a reduced integration of the bone substitute particles. When using a scaffold, the pore size is adjustable in order to optimize the bone ingrowth [3]. Furthermore, an inhomogeneous packing density using particulate bone substitute, that possibly leads to a varying pattern of integration of the granules, can be avoided. However, when analyzing the bone ingrowth into the scaffolds, a pronounced bone ingrowth was obvious at the contact area between the scaffold and the host bone. This finding is in line with the results of Iezzi et al. who observed a higher integration and a pronounced new bone formation into a three-dimensional printed scaffold consisting of hydroxyapatite and tricalcium phosphate at the contact area to the host bone in a sheep model [35,36]. This observation is explainable by the ingrowths of blood vessels which are delivering growths factors enabling cell migration into the scaffold. Furthermore, a study inserting hydroxyapatite scaffolds with a pore size of 300 µm in the maxillary sinus of thirteen patients showed bone ingrowth into the interconnecting pores after three months of healing [37]. This seems to be a promising result in order to shorten the healing time for calcium phosphate cement scaffolds. The pore size with 300 µm is comparable to the pore size used in the presented case with 330 µm and seems to promote bone ingrowth [37].

When using pre-designed scaffolds for sinus floor grafting, an extended access cavity to the sinus is necessary increasing the invasiveness of the surgery. Compared to the crestal approach for sinus floor augmentation, this increased invasiveness is a disadvantage. On the other hand, for the application of the crestal approach, a residual bone height more than five millimeters showed a higher predictability of success [38]. This bone height was not available in the presented case. Thus, the crestal approach would have not been a sufficient option. Likewise, the application of the lateral window technique was described as safe, and post-operative complications are rare when performed by experienced surgeons [39]. This technique was applied in the presented case without any complications which is similar to a case series applying custom milled scaffolds made from hydroxyapatite for bilateral sinus augmentation [30]. The difficulty might be finding the correct localization of the access cavity in order not to oversize the bone lid. However, when virtually designing the scaffolds shape, a cutting guide in the shape of the bone lid can be manufactured using the same data set as it was conducted by Mangano et al. [30]. The application of cutting guides in order to determine resection margins, and the size and shape of free fibular flaps are widely established [40,41,42]. Additionally, the use of cutting guides for the access bone lid when performing enucleation of large mandible cysts was demonstrated as helpful [43].

Considering the healing time, nine months for scaffold integration is within the time period comparable to bone grafting with other substitute materials for sinus floor augmentation [44]. In their systematic review, healing times between 4.5 and 13.5 months were analyzed without a significant additional gain of new bone formation after 9 months [44]. However, regarding the extend of the grafted volumes, we decided to prolongate the healing time in order to obtain a sufficient osseointegrated scaffold. In further investigations, a reduction of the healing time for the scaffold should be examined as a healing time of five months was found to be sufficient considering clinical, histomorphometric and microradiographic parameters using conventional materials [45].

Summarizing, the application of scaffolds made of calcium phosphate cement paste was promising in the presented case. However, the results have to be interpreted with caution. As always in the presentation of single cases it is difficult to predict how the scaffolds would perform in varying individuals. In order to elucidate the performance in a broader population, examinations which include more patients are necessary. Next, scaffolds used for sinus grafting are allowed to heal without any mobility. Thus, our results considering the uneventful healing might be different when applying the scaffolds in the mandible where the scaffolds are exposed to a higher mechanical stress due to muscle tension and the torsion of the mandibular bone itself [46]. This stress might lead to an exposure of the scaffold and, thus, potentially to an infection as it was observed in a study where milled scaffolds made of synthetic hydroxyapatite/beta-tricalcium phosphate were applied [47]. In order to evaluate the influence of the location on scaffold integration, further studies with different grafting sites are necessary. Additionally, only one scaffold design was tested in our case. As the pore size, the strand orientation and the tortuosity have shown to have an influence on the permeability and, thus, on cell migration and proliferation, further examinations seem to be necessary in order to find an ideal design [8,9].

## 5. Conclusions

In the presented case, three-dimensional printed scaffolds made of a calcium phosphate cement paste seemed to be a valuable method for extended sinus grafting enabling a subsequent insertion of dental implants. The workflow in the presented experimental setting proved sufficient from the image acquisition to the production and sterilization of the scaffolds. The clinical application was easily manageable for an experienced surgeon. Furthermore, the healing of the scaffolds and the dental implants was uneventful indicating a sufficient biocompatibility of the material. Summarizing, the presented approach seems to be a promising method for the sinus grafting. However, the application of the scaffolds in a wider clinical setting has to be examined considering the suitability for daily use, the workflow of diagnostic and scaffold production in dentist’s surgery and the patients’ acceptance for this kind of graft. A simplification of the workflow has to be focused on in order to promote the suitability for daily use. Furthermore, variations in the scaffold’s porosity and long-term examinations of the resorption characteristics are needed in order to optimize stability and the bone ingrowth.

## Data Availability

The original datasets analyzed in the current study are available from Dr. Matthias C. Schulz on reasonable request.

## References

[1] Aimar A., Palermo A., Innocenti B. (2019). The Role of 3D Printing in Medical Applications: A State of the Art. J. Health Eng..

[2] Anderson M., Dubey N., Bogie K., Cao C., Li J., Lerchbacker J., Mendonça G., Kauffmann F., Bottino M.C., Kaigler D. (2022). Three-dimensional printing of clinical scale and personalized calcium phosphate scaffolds for alveolar bone reconstruction. Dent. Mater..

[3] Muallah D., Sembdner P., Holtzhausen S., Meissner H., Hutsky A., Ellmann D., Assmann A., Schulz M., Lauer G., Kroschwald L. (2021). Adapting the Pore Size of Individual, 3D-Printed CPC Scaffolds in Maxillofacial Surgery. J. Clin. Med..

[4] Tavelli L., Barootchi S., Rasperini G., Giannobile W.V. (2022). Clinical and patient-reported outcomes of tissue engineering strategies for periodontal and peri-implant reconstruction. Periodontology 2000.

[5] Wach T., Kozakiewicz M. (2020). Fast-Versus Slow-Resorbable Calcium Phosphate Bone Substitute Materials—Texture Analysis after 12 Months of Observation. Materials.

[6] Shi J., Dai W., Gupta A., Zhang B., Wu Z., Zhang Y., Pan L., Wang L. (2022). Frontiers of Hydroxyapatite Composites in Bionic Bone Tissue Engineering. Materials.

[7] Prakasam M., Silvain J.-F., Largeteau A. (2021). Innovative High-Pressure Fabrication Processes for Porous Biomaterials—A Review. Bioengineering.

[8] Kilian D., Holtzhausen S., Groh W., Sembdner P., Czichy C., Lode A., Stelzer R., Gelinsky M. (2023). 3D extrusion printing of density gradients by variation of sinusoidal printing paths for tissue engineering and beyond. Acta Biomater..

[9] Prakoso A.T., Basri H., Adanta D., Yani I., Ammarullah M.I., Akbar I., Ghazali F.A., Syahrom A., Kamarul T. (2023). The Effect of Tortuosity on Permeability of Porous Scaffold. Biomedicines.

[10] Kroczek K., Turek P., Mazur D., Szczygielski J., Filip D., Brodowski R., Balawender K., Przeszłowski Ł., Lewandowski B., Orkisz S. (2022). Characterisation of Selected Materials in Medical Applications. Polymers.

[11] Guvendiren M., Molde J., Soares R.M., Kohn J. (2016). Designing Biomaterials for 3D Printing. ACS Biomater. Sci. Eng..

[12] Latimer J.M., Maekawa S., Yao Y., Wu D.T., Chen M., Giannobile W.V. (2021). Regenerative Medicine Technologies to Treat Dental, Oral, and Craniofacial Defects. Front. Bioeng. Biotechnol..

[13] Zhang D., Wu X., Chen J., Lin K. (2017). The development of collagen based composite scaffolds for bone regeneration. Bioact. Mater..

[14] Yazdanpanah Z., Johnston J.D., Cooper D.M.L., Chen X. (2022). 3D Bioprinted Scaffolds for Bone Tissue Engineering: State-Of-The-Art and Emerging Technologies. Front. Bioeng. Biotechnol..

[15] Ginebra M.-P., Canal C., Espanol M., Pastorino D., Montufar E.B. (2012). Calcium phosphate cements as drug delivery materials. Adv. Drug Deliv. Rev..

[16] Ginebra M.-P., Fernández E., De Maeyer E., Verbeeck R., Boltong M., Ginebra J., Driessens F., Planell J. (1997). Setting Reaction and Hardening of an Apatitic Calcium Phosphate Cement. J. Dent. Res..

[17] Xu H.H.K., Wang P., Wang L., Bao C., Chen Q., Weir M.D., Chow L.C., Zhao L., Zhou X., Reynolds M.A. (2017). Calcium phosphate cements for bone engineering and their biological properties. Bone Res..

[18] Klein R., Tetzlaff R., Weiss C., Schäfer M.-K., Tanner M., Wiedenhöfer B., Grafe I., Meeder P.-J., Noeldge G., Nawroth P.P. (2017). Osteointegration and Resorption of Intravertebral and Extravertebral Calcium Phosphate Cement. Clin. Spine Surg. A Spine Publ..

[19] Reitmaier S., Kovtun A., Schuelke J., Kanter B., Lemm M., Hoess A., Heinemann S., Nies B., Ignatius A. (2017). Strontium (II) and mechanical loading additively augment bone formation in calcium phosphate scaffolds. J. Orthop. Res..

[20] Schumacher M., Gelinsky M. (2015). Strontium modified calcium phosphate cements—Approaches towards targeted stimulation of bone turnover. J. Mater. Chem. B.

[21] Schumacher M., Reither L., Thomas J., Kampschulte M., Gbureck U., Lode A., Gelinsky M. (2017). Calcium phosphate bone cement/mesoporous bioactive glass composites for controlled growth factor delivery. Biomater. Sci..

[22] Cha J.-K., Kim C., Pae H.-C., Lee J.-S., Jung U.-W., Choi S.-H. (2019). Maxillary sinus augmentation using biphasic calcium phosphate: Dimensional stability results after 3–6 years. J. Periodontal Implant. Sci..

[23] Marongiu G., Verona M., Cardoni G., Capone A. (2020). Synthetic Bone Substitutes and Mechanical Devices for the Augmentation of Osteoporotic Proximal Humeral Fractures: A Systematic Review of Clinical Studies. J. Funct. Biomater..

[24] Tatum H. (1986). Maxillary and Sinus Implant Reconstructions. Dent. Clin. N. Am..

[25] Canellas J.V.D.S., Drugos L., Ritto F.G., Fischer R.G., Medeiros P.J.D. (2021). Xenograft materials in maxillary sinus floor elevation surgery: A systematic review with network meta-analyses. Br. J. Oral Maxillofac. Surg..

[26] Lambert F., Léonard A., Drion P., Sourice S., Layrolle P., Rompen E. (2010). Influence of space-filling materials in subantral bone augmentation: Blood clot vs. autogenous bone chips vs. bovine hydroxyapatite. Clin. Oral Implant. Res..

[27] Lundgren S., Cricchio G., Palma V.C., Salata L.A., Sennerby L. (2008). Sinus membrane elevation and simultaneous insertion of dental implants: A new surgical technique in maxillary sinus floor augmentation. Periodontology 2000.

[28] Kirmeier R., Payer M., Wehrschuetz M., Jakse N., Platzer S., Lorenzoni M. (2008). Evaluation of three-dimensional changes after sinus floor augmentation with different grafting materials. Clin. Oral Implant. Res..

[29] Ohayon L., Taschieri S., Friedmann A., Del Fabbro M. (2019). Bone Graft Displacement After Maxillary Sinus Floor Augmentation with or without Covering Barrier Membrane: A Retrospective Computed Tomographic Image Evaluation. Int. J. Oral Maxillofac. Implant..

[30] Mangano F., Zecca P., Pozzi-Taubert S., Macchi A., Ricci M., Luongo G. (2012). Maxillary sinus augmentation using computer-aided design/computer-aided manufacturing (CAD/CAM) technology. Int. J. Med. Robot. Comput. Assist. Surg..

[31] Lode A., Meissner K., Luo Y., Sonntag F., Glorius S., Nies B., Vater C., Despang F., Hanke T., Gelinsky M. (2014). Fabrication of porous scaffolds by three-dimensional plotting of a pasty calcium phosphate bone cement under mild conditions. J. Tissue Eng. Regen. Med..

[32] Donath K., Breuner G. (1982). A method for the study of undecalcified bones and teeth with attached soft tissues*. The Sage-Schliff (sawing and grinding) Technique. J. Oral Pathol. Med..

[33] Nam I.-H.D., Ma Y.-H.D., Jaiswal M.S.D., Hwang J.-M.D., Hwang D.-S.D. (2022). Accuracy of Maxillary Positioning During Orthognathic Surgery: A Comparison of Web-based 3-Dimensional Virtual Surgical Planning and Actual Outcomes. J. Craniofacial Surg..

[34] Putra R.U., Basri H., Prakoso A.T., Chandra H., Ammarullah M.I., Akbar I., Syahrom A., Kamarul T. (2023). Level of Activity Changes Increases the Fatigue Life of the Porous Magnesium Scaffold, as Observed in Dynamic Immersion Tests, over Time. Sustainability.

[35] Reich K.M., Beck F., Heimel P., Lettner S., Redl H., Ulm C., Tangl S. (2022). Bone Graft Packing and Its Association with Bone Regeneration in Maxillary Sinus Floor Augmentations: Histomorphometric Analysis of Human Biopsies. Biology.

[36] Iezzi G., Scarano A., Valbonetti L., Mazzoni S., Furlani M., Mangano C., Muttini A., Raspanti M., Barboni B., Piattelli A. (2021). Biphasic Calcium Phosphate Biomaterials: Stem Cell-Derived Osteoinduction or In Vivo Osteoconduction? Novel Insights in Maxillary Sinus Augmentation by Advanced Imaging. Materials.

[37] Scarano A., Lorusso F., de Oliveira P.S., Padmanabhan S.K., Licciulli A. (2019). Hydroxyapatite Block Produced by Sponge Replica Method: Mechanical, Clinical and Histologic Observations. Materials.

[38] Pjetursson B.E., Lang N.P. (2014). Sinus floor elevation utilizing the transalveolar approach. Periodontology 2000.

[39] Valentini P., Artzi Z. (2022). Sinus augmentation procedure via the lateral window technique—Reducing invasiveness and preventing complications: A narrative review. Periodontology 2000.

[40] Liu Y.-F., Xu L.-W., Zhu H.-Y., Liu S.S.-Y. (2014). Technical procedures for template-guided surgery for mandibular reconstruction based on digital design and manufacturing. Biomed. Eng. Online.

[41] Meyer S., Hirsch J.-M., Leiggener C.S., Msallem B., Sigron G.R., Kunz C., Thieringer F.M. (2020). Fibula Graft Cutting Devices: Are 3D-Printed Cutting Guides More Precise Than a Universal, Reusable Osteotomy Jig?. J. Clin. Med..

[42] Meyer S., Hirsch J.-M., Leiggener C.S., Zeilhofer H.-F., Thieringer F.M. (2019). A simple, effective, universal, and reusable osteotomy tool for jaw reconstructions with microvascular fibula transplants. J. Plast. Reconstr. Aesthetic Surg..

[43] Liu Z., Huang D., Li K., Li H., Liu L. (2021). Precise locating and cutting of the bone lid with a digital template during the treatment of large mandibular cysts: A case series study. J. Cranio-Maxillofac. Surg..

[44] Danesh-Sani S.A., Engebretson S.P., Janal M.N. (2017). Histomorphometric results of different grafting materials and effect of healing time on bone maturation after sinus floor augmentation: A systematic review and meta-analysis. J. Periodontal Res..

[45] Liu Y., Wang J., Chen F., Feng Y., Xie C., Li D. (2020). A reduced healing protocol for sinus floor elevation in a staged approach with deproteinized bovine bone mineral alone: A randomized controlled clinical trial of a 5-month healing in comparison to the 8-month healing. Clin. Implant. Dent. Relat. Res..

[46] Van Eijden T. (2000). Biomechanics of the Mandible. Crit. Rev. Oral Biol. Med..

[47] Mangano C., Luongo G., Luongo F., Lerner H., Margiani B., Admakin O., Mangano F. (2022). Custom-made computer-aided-design/ computer-assisted-manufacturing (CAD/CAM) synthetic bone grafts for alveolar ridge augmentation: A retrospective clinical study with 3 years of follow-up. J. Dent..

